# Association between preoperative peripheral blood mononuclear cell gene expression profiles, early postoperative organ function recovery potential and long-term survival in advanced heart failure patients undergoing mechanical circulatory support

**DOI:** 10.1371/journal.pone.0189420

**Published:** 2017-12-13

**Authors:** Galyna Bondar, Ryan Togashi, Martin Cadeiras, Joanna Schaenman, Richard K. Cheng, Lindsay Masukawa, Josephine Hai, Tra-Mi Bao, Desai Chu, Eleanor Chang, Maral Bakir, Sophie Kupiec-Weglinski, Victoria Groysberg, Tristan Grogan, Joseph Meltzer, Murray Kwon, Maura Rossetti, David Elashoff, Elaine Reed, Pei Pei Ping, Mario C. Deng

**Affiliations:** 1 David Geffen School of Medicine, University of California Los Angeles Medical Center, Los Angeles, California, United States of America; 2 University of Washington Medical Center, Seattle, Washington, United States of America; 3 Cornell University, Ithaca, New York, United States of America; Universidade de Mogi das Cruzes, BRAZIL

## Abstract

**Background:**

Multiorgan dysfunction syndrome contributes to adverse outcomes in advanced heart failure (AdHF) patients after mechanical circulatory support (MCS) implantation and is associated with aberrant leukocyte activity. We tested the hypothesis that preoperative peripheral blood mononuclear cell (PBMC) gene expression profiles (GEP) can predict early postoperative improvement or non-improvement in patients undergoing MCS implantation. We believe this information may be useful in developing prognostic biomarkers.

**Methods & design:**

We conducted a study with 29 patients undergoing MCS-surgery in a tertiary academic medical center from 2012 to 2014. PBMC samples were collected one day before surgery (day -1). Clinical data was collected on day -1 and day 8 postoperatively. Patients were classified by Sequential Organ Failure Assessment score and Model of End-stage Liver Disease Except INR score (measured eight days after surgery): Group I = improving (both scores improved from day -1 to day 8, n = 17) and Group II = not improving (either one or both scores did not improve from day -1 to day 8, n = 12). RNA-sequencing was performed on purified mRNA and analyzed using Next Generation Sequencing Strand. Differentially expressed genes (DEGs) were identified by Mann-Whitney test with Benjamini-Hochberg correction. Preoperative DEGs were used to construct a support vector machine algorithm to predict Group I vs. Group II membership.

**Results:**

Out of 28 MCS-surgery patients alive 8 days postoperatively, one-year survival was 88% in Group I and 27% in Group II. We identified 28 preoperative DEGs between Group I and II, with an average 93% prediction accuracy. Out of 105 DEGs identified preoperatively between year 1 survivors and non-survivors, 12 genes overlapped with the 28 predictive genes.

**Conclusions:**

In AdHF patients following MCS implantation, preoperative PBMC-GEP predicts early changes in organ function scores and correlates with long-term outcomes. Therefore, gene expression lends itself to outcome prediction and warrants further studies in larger longitudinal cohorts.

## Introduction

Heart failure (HF) is a complex clinical syndrome that results from any structural or functional cardiovascular disorder causing a mismatch between demand and supply of oxygenated blood and consecutive failure of the body’s organs. In the United States, HF affects about 6 million persons[1]. HF with reduced ejection fraction (HFrEF) affects 3 million people[2]. The lifetime risk of developing HF for men and women older than 40 years of age is 1 in 5. The death rate remains unacceptably high at approximately 50% within 5 years from time of initial diagnosis. Stage D, or advanced heart failure (AdHF), designates patients with truly refractory HF (estimated at 300,000 persons in the US annually)[2].

AdHF patients may benefit from the following therapeutic options: optimal medical management (OMM) or palliative/hospice care (PC, n = 300,000), mechanical circulatory support (MCS, n = 30,000) or heart transplantation (HTx, n = 3,000)[3]. MCS devices, originally used for patients with AdHF as a bridge-to-transplant or bridge-to-recovery, are now increasingly used as destination (lifelong) therapy and have the potential to outnumber HTx by a factor of 1:10, currently showing an improved survival rate of approximately 80% at 1 year[4].

Because of this success, destination MCS is increasingly being offered to patients with challenging clinical profiles. There is significant patient-to-patient variability for risk of adverse events, including death, after MCS-surgery. The ability to preoperatively predict this risk for the individual AdHF-patient before surgery and the impact of this risk on the associated long-term survival prognosis would be a very important component of clinical decision-making and management. Currently, we have our clinical expertise and validated clinical tools[4–18] for risk prediction.

However, despite our clinical expertise and validated tools, it is not easy to assess this risk and, therefore, make recommendations about which therapy benefits the individual patient most with respect to long-term survival. Often, elderly and frail AdHF patients, if not doing well on OMM, are also at increased risk for organ dysfunction (OD) and death after MCS-surgery. One of the reasons for the current challenges of risk prediction is the difficulty in assessing the degree of frailty and OD in the individual AdHF patient who often suffers from malnutrition, immune dysfunction, and poor infection coping potential.

Preoperative HF-related immunologic impairment is a component of poor outcomes after MCS and HTx, owing to the known associations between increased age, T cell and innate immune cell dysfunction, frailty, increased numbers of terminally differentiated T cells, immune senescence (deficient replicative ability), and immune exhaustion (impaired antigen response)[19–23]. Multi-organ dysfunction syndrome (MOD) is one of the leading causes of morbidity and mortality. It is associated with grossly aberrant immune activation[4–18, 24–26].

None of the current established clinical scoring and prediction tools integrate immune function parameters. They have the tendency to be imprecise in estimating risk among severely ill patients[11, 12], making the therapeutic recommendation with the best survival estimate for the individual patient very difficult. Our central postulate is that OD and patient death after MCS- or HTx-surgery results from innate and adaptive immune cell dysfunction. Therefore, our goal is to use leukocyte immune-biology information to develop a preoperative test, which would precisely predict postoperative outcomes in the individual AdHF patient. We utilized the widely accepted Sequential Organ Failure Assessment (SOFA)[27] and Model of End Stage Liver Disease without INR (MELD-XI) [24, 28, 29] scores as quantitative assessment tools to interpret the PBMC data and to develop a predictive leukocyte biomarker.

In order to achieve this goal, we hypothesize that in AdHF patients undergoing MCS-surgery, HF-related preoperative peripheral blood mononuclear cell (PBMC) gene expression profiles (GEP) correlate with and predict changes of early postoperative organ function status as surrogates for 1 year survival.

In our prior studies, we reported on PBMC GEP time course analyses after MCS-surgery[30–32]. Here, we present data to support our hypotheses that, in AdHF patients undergoing MCS implantation, preoperative differential PBMC-GEP are associated with and are predictive of early postoperative SOFA and MELD-XI score changes, defined as score difference between immediately before surgery to 8 days after surgery as a surrogate marker for long term mortality risk.

We propose to interpret our findings within our novel concept of Functional Recovery Potential (FRP), seen as a person’s quantifiable potential to improve after being exposed to a stressor, such as MCS-surgery.

## Methods & design

### Study design

To address the most pressing clinical problem of MCS-related perioperative MOD[4, 33, 34], we chose to base this analysis on a control population of AdHF-patients undergoing MCS-surgery alone. We conducted a study with 29 AdHF patients undergoing MCS-surgery at UCLA Medical Center between August 2012–2014 under UCLA Medical Institutional Review Board 1approved Protocol Number 12–000351. Written informed consent was obtained from each participant.

#### Clinical management

All study participants were referred to the UCLA Integrated AdHF Program and evaluated for the various therapeutic options, including OMM, MCS, and HTx. All patients were optimized regarding HF therapy, consented to and underwent MCS-therapy according to established guidelines[35, 36], based on the recommendations of the multidisciplinary heart transplant selection committee.

After anesthesia induction, patients were intubated and placed on cardiopulmonary bypass. The type of MCS-device selected depended on the acuity and severity of the heart failure syndrome, as well as patient characteristics[37]. For left ventricular support, patients underwent either Heartmate II (HeartMate II^®^ pumps are valveless, rotary, continuous flow pumps) or HVAD (HeartWare^®^ HVAD pumps are valveless, centrifugal, continuous flow pumps). For biventricular support, patients underwent either Centrimag-BVAD (Centrimag^®^ pumps are valveless, centrifugal, continuous flow pumps that are external to the body), PVAD biventricular assist device (BVAD) (Thoratec^®^ Paracorporeal Ventricular Assist Device (PVAD) pumps each contain two mechanical tilting disk valves) or the t-TAH (the Temporary Total Artificial Heart consists of two artificial ventricles that are used to replace the failing heart).

Various combinations of cardiovascular inotropic and vasoactive drugs were used to support patient’s hemodynamics postoperatively, tailored to the individual requirements. In addition, other temporary organ system support was administered as required (e.g. mechanical ventilation, hemodialysis, blood transfusions, and antibiotic therapy).

#### Clinical phenotyping

Demographic variables were obtained for all patients. Twelve distinct parameters were collected on a daily basis for time-dependent clinical phenotyping of the patient cohort, which included serum bilirubin, serum creatinine, leukocyte count, platelet count, alveolar oxygen pressure, fraction of inspired oxygen (FiO2), mean systemic arterial pressure (MAP), INR (International Normalized Ratio, for prothrombin time), blood glucose, heart rate, respiratory rate, temperature, and the Glasgow Coma Scale (GCS).

Using combinations of these parameters, we also calculated two validated and commonly used composite OD scores, SOFA[27] and MELD-XI[24]. The SOFA score is a validated and widely accepted measure that rates degree of organ failure across six major organ systems (cardiovascular, respiratory, neurological, renal, hepatic, and coagulation). The MELD-XI score is a variation of the MELD score that uses only the bilirubin and creatinine levels, and eliminates the INR, which is typically not interpretable in these patients given the need of anticoagulation.

We also used the Interagency Registry for Mechanically Assisted Circulatory Support (INTERMACS) scoring system, which has been developed to improve patient selection and timing of MCS therapy[4] for preoperative HF-severity assessment. Higher INTERMACS risk categories are considered predictors of worse survival. While INTERMACS identifies clinical outcomes and risk of MOD, it does not provide insights into the underlying immunological mechanisms of disease.

#### Clinical outcome parameter

One of the most significant clinical outcome parameters for AdHF patients undergoing MCS is the probability of organ function improvement from one day before to eight days after surgery.

From a clinical utility perspective, we aim to provide AdHF-patients with the most precise prediction of short- and long-term outcome[38, 39] on either OMM or MCS. Since many AdHF patients have varying recovery potential, we chose a short-term improvement criteria, i.e. 8 days postoperatively, as a surrogate outcome parameter for long-term survival. For these reasons, we chose not to use a static preoperative organ function severity score to develop our biomarker. The most logical clinical parameter is the potential for organ function improvement, which we named the short-term functional recovery potential (FRP). This parameter may identify patients who benefit from aggressive therapies, such as MCS, even if they are very ill.

Therefore, patients were grouped into two organ failure risk strata: Group I = improving (both SOFA and MELD-XI scores improve from day -1 to day 8) and Group II = not improving (SOFA and/or MELD-XI score(s) do not improve from day -1 to day 8). In other words, if the MCS-surgery improves the hemodynamic situation without complications, then the patient’s organ function is expected to recover by postoperative day 5 and clearly by postoperative day 8, which should be reflected in a concordant improvement of SOFA and MELD-XI score, from day -1 to day 8. On the other hand, if SOFA or MELD-XI, or both, scores do not improve from day -1 to day 8, we hypothesize that this problem may potentially impact long-term survival.

#### PBMC sample processing & GEP protocol

PBMC samples were collected one day before surgery (day -1). Clinical data was collected on day -1 and day 8 postoperatively. We chose, based on our successful Allomap^™^ biomarker test development experience[40–43], to focus on the mixed PBMC population.

Eight ml of blood was drawn into a CPT tube (Becton Dickinson, Franklin Lakes, NJ). Peripheral Blood Mononuclear cells (PBMC) from each sample were purified within 2h of phlebotomy. The collected blood was mixed and centrifuged at room temperature (22°C) for 20min at 3000RPM. Two ml of plasma was separated without disturbing the cell layer into an eppendorf tube (Sigma-Aldrich, St. Louis, MO) and stored at -80°C for future experiments. The cell layer was collected, transferred to 15ml conical tubes and re-suspended in cold Phosphate Buffer Saline (PBS) (Sigma-Aldrich, St. Louis, MO) and centrifuged for 20min at 1135RPM at 4°C. The supernatant was aspirated and discharged. The cell pellet was re-suspended in cold PBS, transferred into an eppendorf tube and centrifuged for 20min at 5.6 RPM at 4°C. The supernatant was discharged. The pellet was re-suspended in 0.5 ml RNA Protect Cell Reagent (Qiagen, Valencia, CA) and frozen at -80°C.

#### PBMC transcriptome RNA sequencing

All samples were processed using next-generation RNA sequencing transcriptome analysis at the UCLA Technology Center for Genomics & Bioinformatics. Briefly, the RNA was isolated from the PBMC using RNeasy Mini Kit (Qiagen, Valencia, CA). The quality of the total RNA was assessed using NanoDrop^®^ ND-1000 spectrophotometer (NanoDrop Technologies, Wilmington, DE) and Agilent 2100 Bioanalyzer (Agilent Technologies, Palo Alto, CA) concentration above 50 ng/μl., purity—260/280 ~ 2.0., integrity—RIN > 9.0 and average > 9.5. Then, mRNA library was prepared with Illumina TruSeq RNA kit according to the manufacturer’s instructions (Illumina, San Diego, CA). Library construction consists of random fragmentation of the polyA mRNA, followed by cDNA production using random polymers. The cDNA libraries were quantitated using Qubit and size distribution was checked on Bioanalyzer 2100 (Agilent Technologies, Palo Alto, CA). The library was sequenced on HiSeq 2500. Clusters were generated to yield approximately 725K-825K clusters/mm^2^. Cluster density and quality was determined during the run after the first base addition parameters were assessed. We performed single end sequencing runs to align the cDNA sequences to the reference genome. Generated FASTQ files were transferred to the AdHF Research Data Center where Avadis NGS 1.5 (Agilent, Palo Alto, CA and Strand Scientific, CA) was used to align the raw RNA-Seq FASTQ reads to the reference genome. After RNA extraction, quantification and quality assessment, total mRNA was amplified and sequenced on the whole-genome Illumina HiSeq 2500. Data was then subjected to DeSeq normalization using NGS Strand/Avadis (v2.1 Oct 10, 2014). Batch effects were removed using the ComBat algorithm in R[44].

### Statistical analysis

#### Transcriptome analysis

We were interested in finding the preoperatively differentially expressed genes (DEG) in the GEP of 29 patients, as they correlate to early postoperative organ function improvement as markers for long-term survival outcome. PBMC-genes differentially expressed between Group I and Group II were identified by non-parametric statistics (Mann-Whitney test with Benjamini-Hochberg correction). Since the original False Discovery Rate (FDR) methodology[45] is too conservative for genomics applications and results in a substantial loss of power[46], we used a more relaxed criteria (FDR ≤ 0.1) values as an exploratory guide for which entities to investigate further. Only those genes with fold change of at least 2.0 were included in the analysis. Biological significance was assessed using gene ontology, pathway analysis and via GeneCards database.

#### Prediction model building and testing

To classify postoperative Group I vs. Group II, we constructed a PBMC-GEP prediction model on preoperative day -1 gene expression data using the support vector machine (SVM) algorithm. Out of 29 samples, 20 were randomly selected to build the model and the remaining 9 samples, stratified by membership in Group I or Group II, were used to test the model. The prediction model was tested on 25 repetitions with random sampling.

#### Quantitative Real-time polymerase chain reaction (RT-qPCR) validation

NGS data were validated by Quantitative PCR obtained from PBMC of 6 samples taken across Group I (n = 3) and Group II (n = 3). Total RNA from PBMC were purified using RNeasy Mini Kit (Qiagen, Valencia, CA). CDNA was synthesized with iScript supermix for RT-qPCR (BioRad, Hercules, CA). RT-qPCR analysis was carried out with iTaq SYBR green supermix (BioRad, Hercules, CA) on the 7500 Fast Real-time PCR system (Applied Biosystems, Foster City, CA). GAPDH levels were used as an internal control for RT-qPCR. Sequences of the primer pairs used were as follows: *GAPDH-f*: *CCACTCCTCCACCTTTGAC*; *GAPDH-r*: *ACCCTGTTGCTGTAGCCA*; *KIR2DL4-f*: *ACCCACTGCCTGTTTCTGTC*; *KIR2DL4-r*: *ATCACAGCATGCAGGTGTCT*; *NAPSA-f*: *CAGGACACCTGGGTTCACAC*; *NAPSA-r*: *GGTTGGACTCGATGAAGAGG*; *BATF2-f*: *AAAGGCAGCTGAAGAAGCAG*; *BATF2-r*: *TCTTTTTCCAGAGACTCGTGC*; *ANKRD22-f*: *CTCAGCCAGGAAGGATTTTG*; *ANKRD22-r*: *TGATAGGCTGCTTGGCAGAT*.

## Results

### Clinical profiles and outcomes

#### Pre-, intra- and postoperative clinical profiles and long-term survival

**Fig 1:**
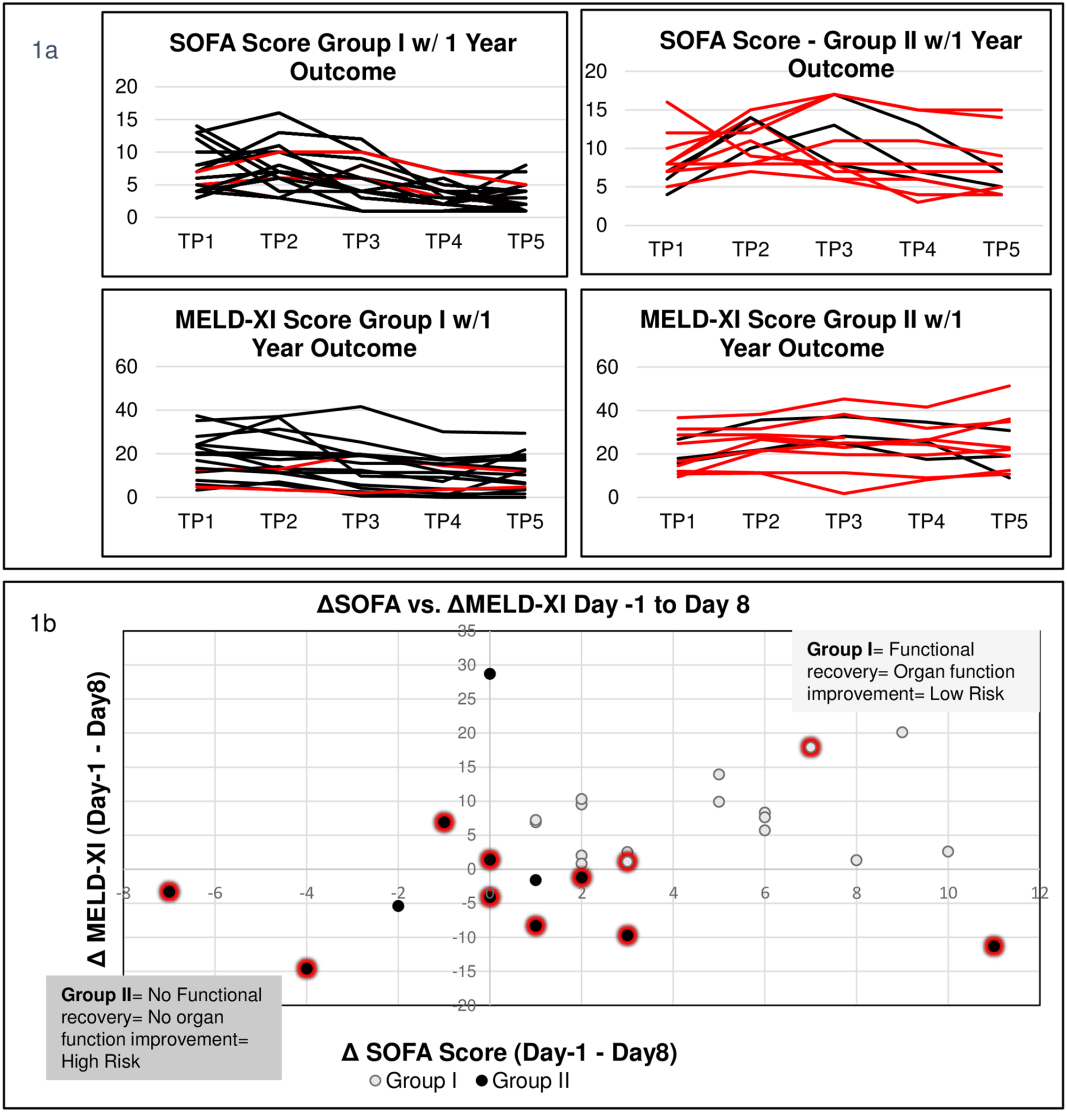
Organ function and outcomes. (A) Organ function and outcomes of 29 patients across five time points. Out of 29 AdHF-patients undergoing MCS-surgery, 17 patients had organ function improvement from preoperative day -1 (TP1) to day 8 (TP5) (Group I) and 12 patients had no organ function improvement (Group II). Each black line represents one 1-year survivor while each red line represents one 1-year non-survivor. In each group, non-survivors are shown in red. (B) Out of 29 AdHF-patients undergoing MCS-surgery, 17 patients improved (Group I, upper right quadrant) and 12 patients did not improve (Group II, remaining 3 quadrants) from day -1 (TP1) to day 8 (TP5). Each large dark bullet represents one patient who died within one year. Absence of improvement of either score was associated with reduced 1-year survival.

**Fig 2:**
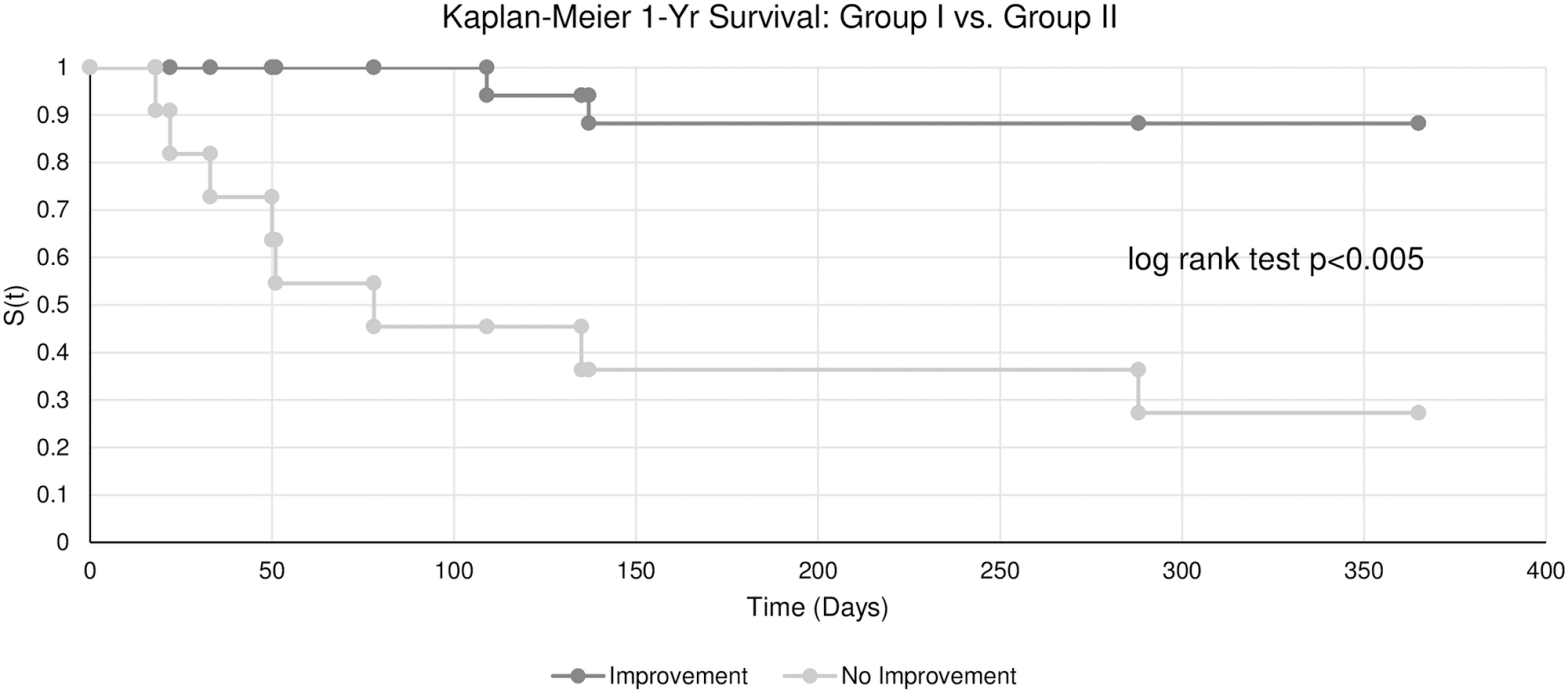
Kaplan-Meier 1-year survival in Group I vs. Group II. In the 17 patients who improved (Group I = Functional recovery = Organ function improving = Low Risk) vs. the 11 patients who did not improve (Group II = No functional recovery = Organ function not improving = High Risk), the time-to-event Kaplan-Meier survival analysis suggested that the significantly elevated risk (log rank test p = 0.00182) of death in Group II continued over the 1-year period following MCS-surgery.

Out of 29 patients, 17 were preoperatively in INTERMACS class 1–2 (a state of critical cardiogenic shock or progressively declining on inotropic support), while the remaining 12 patients were in INTERMACS class 3–4 (inotrope dependent or resting symptoms)[4]. Characteristics of the patients are shown in Table 1. The SOFA and MELD-XI OD trajectory for each group is summarized in Fig 1A. The same data in terms of amount of improvement is shown in Fig 1B. One-year survival in Group I was 15/17 and in Group II 3/11, indicating lower risk in Group I (Fisher’s Exact Test p<0.005). Importantly, the time-to-event Kaplan-Meier survival analysis suggested that the significantly elevated risk of death in Group II vs. Group I continued over the 1-year period following MCS-surgery (log rank p = 0.00182; Fig 2).

**Table 1:** Demographics on 29 AdHF-patients undergoing MCS-surgery.

DEMOGRAPHICS			CLINICAL PARAMETERS								TP1			TP5		OBSERVED		PRE-OP		INTRA-OP		POST-OP
AGE	GENDER	UNDERLYING DISEASE	Bilirubin (mg/dL)	Creatine (mg/dL)	INR	WBC	Heart Rate	Respiratory Rate	Glucose	MCS TYPE	INTERMACS	SOFA	MELDXI	SOFA	MELDXI	GROUP	OUTCOME AFTER 1 YEAR	ECHO^a^	Inotropes^b^	Cardio-pulmonary Bypass Time^c^	≥4 Blood Transfusions^d^	Cause of Death
59	Female	NICM	1.15	1.80	1.1	7.63	85	20	144	HMII	1–2	4	20.4	2	18.4	I	Alive	1	2	1	2	N/A
38	Female	PPCM	1.60	0.57	1.7	6.50	121	22	113	HMII/ CMAG-RVAD	3–4	5	3.3	4	-3.6	I	Alive	2	2	1	1	N/A
25	Female	NICM	1.00	1.40	1.1	7.60	94	20	94	HMII	3–4	4	13.4	2	3.9	I	Alive	1	2	1	1	N/A
43	Male	ICM	1.80	1.73	1.2	15.31	122	17	203	HMII	3–4	12	23.1	4	21.8	I	Alive	1	3	1	1	N/A
58	Male	NICM	2.45	0.77	2.2	17.55	50	17	161	HMII/ CMAG-RVAD	3–4	7	11.5	2	1.6	I	Alive	2	3	2	1	N/A
75	Male	ICM	2.70	3.37	1.2	6.18	83	15	166	HMII	1–2	14	37.4	5	17.3	I	Alive	1	3	1	1	N/A
65	Male	NICM	2.30	1.33	1.5	10.57	93	13	212	HMII	1–2	8	20.5	2	12.9	I	Alive	1	3	2	1	N/A
43	Female	NICM	1.50	1.60	1.6	12.24	118	18	208	HMII/ CMAG-RVAD	1–2	3	17	1	6.7	I	Alive	2	0	2	1	N/A
30	Female	PPCM	1.40	0.70	1.6	15.80	108	17	162	HMII	3–4	4	5.9	1	3.4	I	Alive	2	1	2	1	N/A
69	Male	NICM	2.00	2.20	1.7	8.44	111	24	235	HMII	1–2	10	27.9	4	19.6	I	Alive	2	3	1	2	N/A
67	Female	NICM	0.90	2.50	2.5	15.19	80	15	131	HVAD	3–4	6	24.2	1	10.3	I	Alive	2	2	1	1	N/A
38	Female	NICM	1.60	1.00	0.9	12.60	99	24	91	PVAD BVAD	1–2	5	12.9	2	11.8	I	Dead	2	0	2	1	MOD
61	Male	NICM	4.30	2.40	1.1	10.03	125	16	122	TAH	1–2	13	35.1	7	29.4	I	Alive	2	2	2	1	N/A
37	Male	NICM	2.90	1.50	1.5	8.42	90	18	131	PVAD BVAD	1–2	8	24.2	1	6.3	I	Alive	2	2	2	1	N/A
46	Male	ChemoCM	0.70	2.80	1.7	23.15	63	22	130	HMII	1–2	13	19.7	3	17.1	I	Alive	1	3	1	2	N/A
50	Female	ICM	0.40	1.30	1.1	8.39	101	19	137	HMII	3–4	3	7.8	8	12.1	I	Alive	2	0	2	1	N/A
74	Male	ICM	0.40	1.00	1.0	5.75	59	20	87	HMII	3–4	7	4.8	5	4.0	I	Dead	2	2	1	1	GI bleed
66	Male	NICM	1.80	2.85	1.4	15.00	81	20	288	HMII/ CMAG-RVAD	1–2	8	31.5	15	34.8	II	Dead	1	2	2	2	MOD
62	Male	ICM	1.30	2.20	1.1	11.58	100	intub	143	HMII	1–2	16	24.8	5	36.1	II	Dead	2	3	1	1	MOD
66	Male	ICM	2.10	2.00	1.2	16.52	101	38	245	HMII	1–2	7	26.7	7	30.8	II	Alive	2	0	1	2	N/A
62	Male	NCIM	1.43	1.80	1.3	14.68	129	20	198	HMII	1–2	6	17.9	4	19.1	II	Alive	2	0	1	2	N/A
72	Male	ICM	2.00	0.80	1.1	9.81	75	20	103	HMII/ CMAG-RVAD	3–4	5	10.8	4	12.4	II	Dead	2	0	2	2	MOD
62	Male	ICM	6.60	2.20	1.4	14.05	85	20	118	CMAG-LVAD	1–2	10	36.7	14	51.3	II	Dead	1	1	1	1	MOD
63	Male	ICM	2.00	2.30	1.5	5.88	112	19	139	ECMO	1–2	12	28.7			II	Dead	2	2	1	2	MOD
81	Male	ICM	2.00	1.00	1.2	8.53	70	20	91	HMII	3–4	8	14.6	7	22.9	II	Dead	1	2	2	2	MOD
66	Male	NICM	1.10	2.00	1.1	9.36	80	18	247	HMII	1–2	4	15.9	5	9	II	Alive	1	3	2	1	N/A
49	Male	NICM	0.50	1.70	1.3	6.52	83	19	132	HVAD	3–4	8	12.1	8	10.7	II	Dead	1	2	1	1	Sepsis
38	Male	NICM	1.00	1.00	1.5	11.02	92	38	98	TAH	1–2	7	9.4	4	19.1	II	Dead	2	0	2	1	MOD
68	Male	ICM	1.20	1.70	1.1	7.78	90	22	224	HMII	3–4	7	16.6	9	22	II	Dead	1	1	2	1	MOD

TP = time point, INR = international normalized ratio, WBC = white blood cells, NICM = nonischemic dilated cardiomyopathy, PPCM = peripartum cardiomyopathy, ICM = ischemic cardiomyopathy, ChemoCM = chemotherapy-induced cardiomyopathy, HM II = Heartmate II, CMAG = Centrimag, LVAD = Left ventricular assist device, RVAD = right ventricular assist device, BVAD = biventricular assist device, HVAD = Heartware LVAD, TAH = Total Artificial Heart, ECMO = extracorporeal membrane oxygenator, intub = intubate, GI = gastrointestinal, MOD = multi-organ dysfunction. GROUP: Organ function changes of SOFA-score and MELD-XI score from preoperative day -1 (TP1) to postoperative day 8 (TP5) (Group I: Improvement vs. Group II: Non improvement), A = Alive, D = Dead. The 63-year-old male patient who died before TP5 is missing TP-related SOFA- and MELD-XI data.

^a^ = Preoperative echocardiographic criteria: 1 = normal or mildly reduced right ventricular function, 2 = moderately or severely reduced right ventricular function.

^b^ = Preoperative inotrope support levels: 0 = no inotrope, 1 = 1 inotrope, 2 = 2 inotropes, 3 = ≥3 inotropes or MCS (e.g. VA ECMO).

^c^ = Intraoperative cardiopulmonary bypass (CPB) time: 1 = <median shorter CPB time or no CPB (e.g. minimally invasive LVAD-placement), 2 = ≥median CPB time.

^d^ = Intraoperative bleeding severity as defined per INTERMACS criteria (≥4 RBC per any 24h period during the first 8 postoperative days): 1 = without major intraoperative bleeding, 2 = with major intraoperative bleeding.

Neither correlation between preoperative clinical variables (i.e. INTERMACS class, SOFA median score, MELD-XI median score, and Seattle Heart Failure Model, excluding respiratory rate) nor intra/postoperative clinical variables predict Group I versus Group II membership or year 1 survival status (Table 1). We grouped preoperative right ventricular function, defined by echocardiographic criteria, into two groups: normal to mildly reduced right ventricular function (n = 12) and moderately to severely reduced right ventricular function (n = 17). The chi-square p-value for postoperative Group I versus II membership was non-significant (p = 0.42). We grouped preoperative inotrope support levels into the following categories: no inotrope (n = 7), 1 inotrope (n = 3), 2 inotropes (n = 11), ≥3 inotropes or MCS (e.g. VA ECMO) (n = 8). The chi-square p-value for postoperative Group I versus II membership was non-significant (p = 0.61). Additional preoperative clinical information data (i.e. bilirubin, creatinine, international normalized ratio, white blood cells, heart rate, and glucose level, all non-significant chi-square p-value) (respiratory rate, p = 0.03) are also summarized in Table 1. None of the 29 patients had a clinical infection episode on the day prior to MCS surgery.

The intraoperative median cardiopulmonary bypass (CPB) time was 107min (25%/75%: 75min/145min). We categorized patient CPB time into two groups: patients with no CPB (e.g. minimally invasive LVAD-placement) or CPB time shorter than the median time (n = 15) and patients with CPB time equal to or longer than the median time (n = 14). The chi-square p-value for Group I versus II membership was non-significant (p = 0.51). Additionally, the group without major intraoperative bleeding (n = 20) was compared to those patients with major bleeding (n = 9). Bleeding severity was defined per INTERMACS criteria as greater than or equal to 4 RBC per any 24h period during the first 8 postoperative days. The chi-square p-value for postoperative Group I versus II membership was non-significant (p = 0.06).

Out of 11 patients who died postoperatively, 9 patients died from MOD, 1 patient from gastro-intestinal hemorrhage and 1 patient from sepsis.

### Correlation between preoperative PBMC-transcriptome and clinical outcomes

#### PBMC-transcriptome and clinical course

Out of 29 patients undergoing MCS-surgery, 17 were in Group I and 12 in Group II. Twenty-eight MCS-surgery patients were alive 8 days postoperatively. Since our study explored how the preoperative PBMC-transcriptome can predict postoperative clinical outcomes, we restricted our analysis to the relationship between preoperative day -1 PBMC data and change of clinical data from preoperative day -1 to postoperative day 8. This project is based on our previously published studies that characterized the postoperative correlation between PBMC GEP and clinical parameters[30, 31], as well as our time-course analysis of the correlation between PBMC GEP module eigengenome and clinical parameters[32].

#### Preoperative PBMC-transcriptome and early postoperative organ function changes

**Fig 3:**
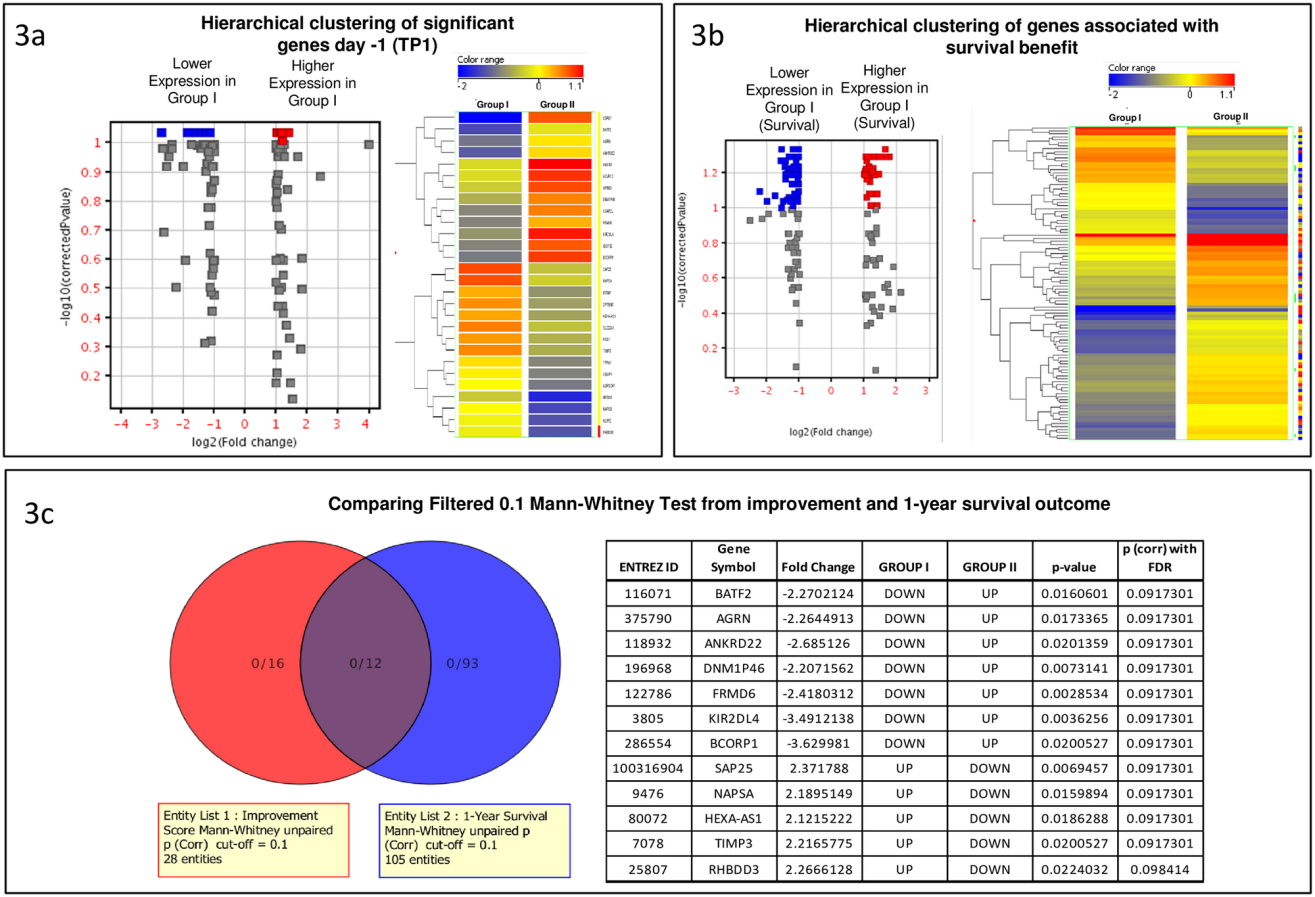
Overlap of significant genes associated with organ function improvement and survival benefit. (A) Hierarchical clustering of significant genes day -1 (TP1). Left: The Volcano plot of 28 genes, which are differentially expressed between Group I and Group II. Right: Hierarchical clustering of the 28 candidate genes for the prediction test demonstrates the differential gene expression between Group I and Group II. (B) Hierarchical clustering of genes associated with survival benefit. Left: The Volcano plot of 105 genes, which are differentially expressed between Group I and Group II. Right: Hierarchical clustering of the 105 candidate genes for the prediction test demonstrates the differential gene expression between Group I = Survival, Group II = Non-survival. (C) Overlap genes from both improvement group and 1-year survival outcome. Left: Venn-Diagram shows the 28 DEGs identified in the comparison by improvement score (red) and the Right shows the 105 DEGs identified by comparing 1-Year Survival (blue). Twelve DEGs were shared across the two comparisons. Right: The 12 overlap genes (see text for details).

In order to identify day -1 transcripts related to organ function change, the entire set of mRNA transcripts (36,938) was filtered (20th-100th percentile)[44]. Of the resulting 26,571 entities, only those with a fold change of at least 2.0 (123 transcripts) were retained for statistical analysis with the unpaired Mann-Whitney test and Benjamini-Hochberg correction analysis (FDR = 0.1). After these filtering steps, 28 genes were identified as differentially expressed between the two groups on day -1 (Fig 3A, Table 2).

**Table 2:** Pre-operative predictive gene list performance and biological summary.

ENTREZ ID	Gene Symbol	Fold Change	GROUP I	GROUP II	P-value	P (corr) with FDR	Gene Summary
8287	*USP9Y*	-6.442405	DOWN	UP	0.01792041	0.09173012	*USP9Y* is associated to Ubiquitin-Proteasome Dependent Proteolysis, and essential component of TGF-beta/BMP signaling cascade. Within nondiabetic heart failure-associated genes with ischemic cardiomyopathy, it was shown to have a high degree of upregulation.
116071	*BATF2*	-2.2702124	DOWN	UP	0.01606008	0.09173012	*BATF2* controls the differentiation of lineage-specific cells in the immune system. Following infection, participates in the differentiation of CD8(+) thymic conventional dendritic cells in the immune system. Selectively suppresses *CYR61/CCN1* transcription and hence blocks the downstream cell proliferation signals produced by *CYR61* and inhibits *CYR61*-induced anchorage-independent growth and invasion in several cancer types; interferons (IFNs), apart from their function as antiviral infection agents, exert a variety of inhibitory effects on cell growth, apoptosis, and angiogenesis. IFNs induce growth inhibition by a variety of pathways that involve many IFN-stimulated genes *BATF2* is one of these genes and can be induced by IFNb, which indicates that *BATF2* may be a key component involved in IFN signaling.
375790	*AGRN*	-2.2644913	DOWN	UP	0.0173365	0.09173012	*AGRN* is responsible for the maintenance of neuromuscular junction and directs key events in postsynaptic differentiation.
118932	*ANKRD22*	-2.685126	DOWN	UP	0.02013588	0.09173012	*ANKRD22* shows the highest upregulation with a value of 3.06 in the RT-qPCR analysis in finding diagnostic biomarkers in Pancreatic Adenocarcinoma Patients. The function of *ANKRD22* remains unknown, but it has been patented by Rosenthal et al. as a possible biomarker for several types of cancer and by Brichard et al. for identification of the patient response to cancer immunotherapy.
83872	*HMCN1*	-2.608948	DOWN	UP	0.00819846	0.09173012	*HMCN1* encodes a large extracellular member of the immunoglobulin superfamily it is associated with age-related and postpartum depression.
130399	*ACVR1C*	-2.2228224	DOWN	UP	0.00538353	0.09173012	*ACVR1C* is a type I receptor for the TGFB, plays a role in cell differentiation, growth arrest and apoptosis.
81491	*GPR63*	-2.2556078	DOWN	UP	0.00260203	0.09173012	*GPR63* is a G-protein coupled receptor activity and plays a role in brain function.
196968	*DNM1P46*	-2.2071562	DOWN	UP	0.00731407	0.09173012	*DNM1P46* is a pseudogene. Although not fully functional, pseudogenes may be functional, similar to other kinds of noncoding DNA, which can perform regulatory functions.
150468	*CKAP2L*	-2.7842844	DOWN	UP	0.00412917	0.09173012	*CKAP2L* is a microtubule-associated protein.
122786	*FRMD6*	-2.4180312	DOWN	UP	0.00285335	0.09173012	*FRMD6* is a Protein Coding gene. Among its related pathways are cytoskeletal signaling and hippo signaling pathway.
3805	*KIR2DL4*	-3.4912138	DOWN	UP	0.00362563	0.09173012	*KIR2DL4* is part of the killer cell immunoglobulin-like receptors, which are transmembrane glycoproteins expressed by natural killer cells and subsets of T cells. Inhibits the activity of NK cells thus preventing cell lysis. Unlike classic HLA class I molecules, HLA-G does not seem to possess significant immune stimulatory functions, and even responses directed against allogeneic HLA-G have not been reported. HLA-G, however, possesses the capability common to HLA class I molecules, to bind inhibitory receptors (Fig 1C). Three HLA-G receptors have been described: *ILT2/CD85j/LILRB1 (ILT2)*, *ILT4/ CD85d/LILRB2 (ILT4)*, *and KIR2DL4/CD158d (KIR2DL4)*.
285313	*IGSF10*	-3.154924	DOWN	UP	0.00996186	0.09173012	*IGSF10* (Immunoglobulin Superfamily Member 10) is a protein coding gene.
286554	*BCORP1*	-3.629981	DOWN	UP	0.0200527	0.09173012	*BCORP1* is a pseudogene. Although not fully functional, pseudogenes may be functional, similar to other kinds of noncoding DNA, which can perform regulatory functions.
100316904	*SAP25*	2.371788	UP	DOWN	0.00694567	0.09173012	*SAP25* is a new member of the growing family of nucleocytoplasmic shuttling proteins that are located in promyelocytic leukemia (PML) nuclear bodies. PML nuclear bodies are implicated in diverse cellular functions such as gene regulation, apoptosis, senescence, DNA repair, and antiviral response. Involved in the transcriptional repression.
9476	*NAPSA*	2.1895149	UP	DOWN	0.01598942	0.09173012	*NAPSA* is a pronapsin, which may have considerable diagnostic value as a marker for primary lung cancer. In contrast, the pronapsin B gene, which lacks an in-frame stop codon and so may be a transcribed pseudogene, is expressed at comparable levels in normal human spleen, thymus, cytotoxic and helper T-lymphocytes, natural killer (NK) cells and B-lymphocytes; may also function in protein catabolism.
161247	*FIT1*	2.272873	UP	DOWN	0.00938506	0.09173012	*FIT1* in skeletal muscle and *FIT2* in adipose, it is interesting to speculate that *FIT1* might be essential for the rapid oxidation of FAs stored as TG in LDs while *FIT2* is required for the long-term storage of TG in adipocytes. Plays an important role in lipid droplet accumulation.
51332	*SPTBN5*	2.3029516	UP	DOWN	0.01434127	0.09173012	*SPTBN5* is related to pathways of Interleukin-3, 5 and GM-CSF signaling and Signaling by GPCR.
80072	*HEXA-AS1*	2.1215222	UP	DOWN	0.01862884	0.09173012	*SPTBN5* (Spectrin Beta, Non-Erythrocytic 5) is a protein coding gene. *HEXA-AS1* (HEXA Antisense RNA 1) is an RNA Gene, and is affiliated with the antisense RNA class.
6580	*SLC22A1*	2.033342	UP	DOWN	0.0173365	0.09173012	*SLC22A1* (Solute Carrier Family 22 Member 1) is a protein coding gene. Plays a critical for elimination of many endogenous small organic cations as well as a wide array of drugs and environmental toxins.
79363	*RSG1*	2.0549338	UP	DOWN	0.00735565	0.09173012	Differential expression *of ABCA1*, *RSG1* and *ADBR2* was replicated in monocyte gene expression in patients with early onset coronary artery disease (CAD). These three genes identified expressed differently in CAD cases which might play a role in the pathogenesis of atherosclerotic vascular disease. Potential effector of the planar cell polarity signaling pathway.
7078	*TIMP3*	2.2165775	UP	DOWN	0.0200527	0.09173012	*TIMP3* blocks the binding of VEGF to VEGF receptor-2 and inhibits downstream signaling and angiogenesis. This property seems to be independent of its MMP-inhibitory activity, indicating a new function for this molecule. Complexes with metalloproteinases (such as collagenases) and irreversibly inactivates them by binding to their catalytic zinc cofactor. Diseases associated with *TIMP3* include Sorsby Fundus Dystrophy and pseudoinflammatory.
131601	*TPRA1*	2.0833867	UP	DOWN	0.01276787	0.09173012	*TPRA1* whose physiological functions are unknown, was first cloned as a GLP-1 receptor homolog in 3T3-L1 adipocytes and is also expressed in tissues whose development requires Hh signaling, including heart, brain, lung, pancreas, and muscle.
752014	*CEMP1*	2.0399396	UP	DOWN	0.01435211	0.09173012	*CEMP1* (Cementum Protein 1) is a Protein Coding gene. Diseases associated with *CEMP1* include coccidiosis.
79058	*ASPSCR1*	2.0533528	UP	DOWN	0.01435211	0.09173012	*ASPSCR1* encodes a protein that contains a UBX domain and interacts with glucose transporter type 4 (GLUT4). This protein is a tether, which sequesters the *GLUT4* in intracellular vesicles in muscle and fat cells in the absence of insulin, and redistributes the *GLUT4* to the plasma membrane within minutes of insulin stimulation.
113655	*MFSD3*	2.3885236	UP	DOWN	0.00672813	0.09173012	Membrane-bound solute carriers (SLCs) are essential as they maintain several physiological functions, such as nutrient uptake, ion transport and waste removal. The SLC family comprise about 400 transporters, and two new putative family members were identified, major facilitator superfamily domain containing 1 (*MFSD1*) and 3 (*MFSD3*).
256236	*NAPSB*	2.6573431	UP	DOWN	0.01610727	0.09173012	*NAPSB* is a pseudogene. Although not fully functional, pseudogenes may be functional, similar to other kinds of noncoding DNA, which can perform regulatory functions.
55655	*NLRP2*	2.3330774	UP	DOWN	0.01434127	0.09173012	*NLRP2* suppresses TNF- and CD40-induced *NFKB1* activity at the level of the IKK complex, by inhibiting *NFKBIA* degradation induced by TNF. When associated with *PYCARD*, activates *CASP1*, leading to the secretion of mature pro-inflammatory cytokine *IL1B*. May be a component of the inflammasome, a protein complex which also includes *PYCARD*, CARD8 and CASP1 and whose function would be the activation of pro-inflammatory caspases.
25807	*RHBDD3*	2.2666128	UP	DOWN	0.02240318	0.09841397	*RHBDD3*, a member of the rhomboid family of proteases, suppressed the activation of DCs and production of interleukin 6 (IL-6) triggered by Toll-like receptors (TLRs). *RHBDD3*-deficient mice spontaneously developed autoimmune diseases characterized by an increased abundance of the TH17 subset of helper T cells and decreased number of regulatory T cells due to the increase in *IL-6* from DCs."

#### Preoperative PBMC-transcriptome and 1-year outcome

Eighteen out of 29 patients were alive after 1 year while 11/29 died during year 1. The causes of death are summarized in Table 1. The preoperative GEP was different in year 1 survivors and non-survivors. The filtered 25,319 entities were analyzed using 2.0 fold change criteria. The 177 differentially expressed genes were analyzed by unpaired Mann-Whitney test with Benjamini-Hochberg correction, resulting in 105 transcripts (FDR = 0.1). Hierarchical clustering was used on the 105 differentially expressed genes for the year 1 survival patients (Fig 3B). Out of these genes, 12 overlap with the 28 genes that correlated with day 8 organ function improvement (Fig 3C, Table 2).

Out of the 28 genes that were differentially expressed between the two groups (Group I vs Group II membership) on postoperative day 8, 12 genes overlapped with 1-year survival status (blue rows).

### PBMC-GEP prediction model development

#### Clinical profiles and outcome correlation

**Fig 4:**
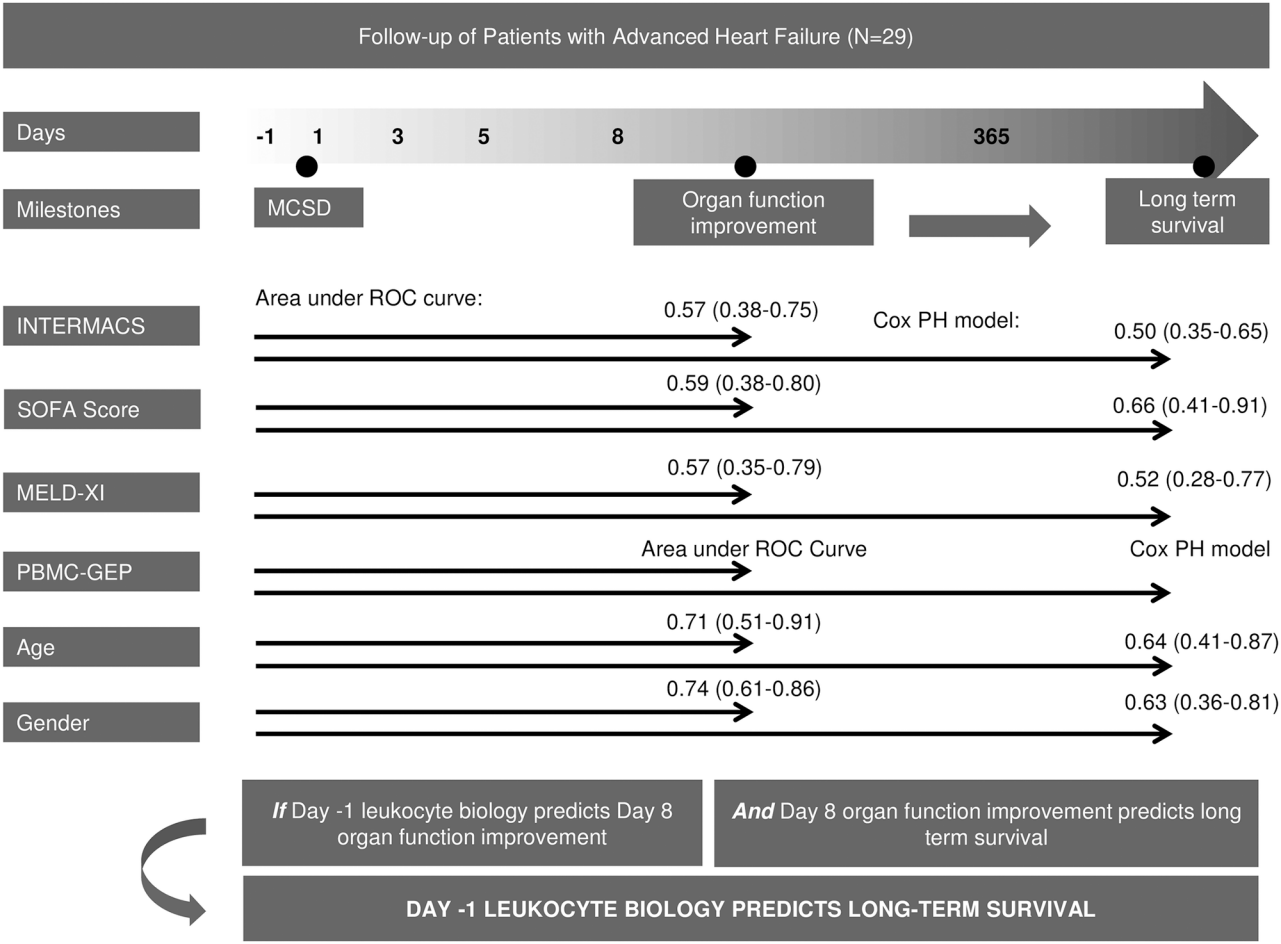
Prediction biomarker development rationale. Preoperative clinical heart failure/organ function severity scores (INTERMACS class, SOFA median score, MELD-XI median score) and demographics (age, gender) did not reliably discriminate postoperative organ function improvement (ROC, 95% confidence interval) and long term survival (Cox Proportional Hazard Model, 95% confidence interval). In contrast, the PBMC-GEP correlates well with postoperative organ function improvement and long term survival.

Neither preoperative clinical variables including INTERMACS[4] class, SOFA median score, MELD-XI median score, and Seattle Heart Failure Model (SHFM) nor intra/postoperative clinical variables (Table 1) (except for respratory rate) predict Group I versus Group II membership nor year 1 survival status. On day 8, 17 patients had organ function improvement (Group I) and 12 patients had no organ function improvement (Group II), with one died on postoperative day 3. Nine patients in INTERMACS class 1–2 preoperatively improved at day 8, while 8 patients did not improve (Fisher’s Exact Test p<0.005). Eight patients in INTERMACS class 3–4 improved while 4 did not improve (Fig 4). The inefficiency of clinical scores in correlating with OD in critically ill AdHF patients[10] supports our rationale in developing a preoperative biomarker prediction test.

#### Prediction of early postoperative organ function changes

We built a model using the SVM algorithm by randomly selecting 20 samples out of 29 total, stratified by membership in Group I versus Group II. To test the model, the remaining 9 samples were stratified by membership in Group I or Group II. An average prediction accuracy of 93% (range: 78–100%) was achieved after running the model building process 25 times (Table 3).

**Table 3:** Prediction of organ function improvement Group I vs II.

	Run Accuracy %		Run Accuracy %
PM1	100	PM14	100
PM2	100	PM15	89
PM3	100	PM16	100
PM4	89	PM17	89
PM5	89	PM18	100
PM6	78	PM19	100
PM7	100	PM20	89
PM8	89	PM21	89
PM9	89	PM22	100
PM10	89	PM23	100
PM11	100	PM24	89
PM12	100	PM25	89
PM13	100	Average:	94

Out of 29 samples, 20 were randomly selected, stratified by membership in Group I or Group II, were used to build the model and the remaining 9 samples were used to test the model.

#### RT-qPCR validation

To validate the NGS results in this study, we performed a limited RT-qPCR experiment to assay the 4 highest ranked genes (by statistical significance and correlation between Group I and Group II expression levels). Results show that 2 out of 4 genes (*KIR2DL4*, *BATF2*) concordantly correlated between NGS and RT-qPCR expression levels, showing downregulation in Group I and upregulation in Group II. Those 2 genes therefore might become candidates for the prognostic test development. The RT-qPCR results of *ANKRD22* and *NAPSA* expression level showed an equivocal relationship to the NGS results. We attributed this discrepancy to the difference in method. This result is in agreement with the internal validation during the Allomap^™^ test development, in which 68 out of 252 candidate genes discovered by high-throughput technology were confirmed by concordant expression changes using RT-qPCR. Therefore, these 68 genes were retained for further Allomap^™^ test development.

## Discussion

We present data to support our hypotheses that in AdHF patients undergoing MCS implantation, preoperative differential PBMC-GEP are associated with and are predictive of early postoperative SOFA and MELD-XI score changes. We defined these clinical parameters as the difference in score between one day before surgery and 8 days after surgery as a surrogate marker for long-term mortality risk. Our studies show the set of 28 genes derived from preoperative PBMC GEP is predictive of early postoperative improvement or non-improvement of SOFA and MELD-XI scores. Out of the 28 preoperative genes, the following 12 genes are of specific biological interest due to their overlap in differentiating early postoperative organ function improvement and year 1 survivor status. (Fig 3, Table 2).

### Potential biological implications of overlapping genes

#### Hypothetical mechanisms of up-regulated genes in non-improvement of SOFA score and MELD-XI score and year 1 non-survivors

*BATF2* belongs to a class of transcription factors that regulate various immunological functions and control the development and differentiation of immune cells. Functional studies demonstrated a predominant role for *BATF2* in controlling Th2 cell functions and lineage development of T lymphocytes. Following infection, *BATF2* participates in the development of and differentiation of CD8 (+) thymic conventional dendritic cells in the immune system[47]. *BATF2* plays a key component involved in IFN signaling and positive regulation of immune responses by altering expression of cytokines and chemokines. Therefore, it possibly maintains the balance in inflammatory processes. *BATF2* is an essential transcription factor for gene regulation and effector functions in classical macrophage activation[48]. *AGRIN* is a gene with a ubiquitous role and is evolutionarily conserved in the extracellular matrix (ECM)[49]. Its intracellular processes include proliferation, apoptosis, migration, motility, autophagy, angiogenesis, tumorigenesis, and immunological responses[50, 51]. *AGRIN* interacts with the α/β-dystroglycan receptor in the formation of immunological synapses with lymphocytes and aids in activation[52] as well as maintaining monocyte cell survival downstream in an α-dystroglycan dependent manner[53]. The AGRIN LG3 domain has been used as a biomarker for detection of prematurely ruptured fetal membranes[54]. *ANKR22*, involved in the lipid modification of proteins[55], has been patented as a possible biomarker for several types of cancers[56, 57] to identify patient responses to cancer immunotherapy. *FRMD6* has been linked to various complex diseases, such as asthma, Alzheimer’s disease, and lung cancer. It plays a critical role in regulating both cell proliferation and apoptosis, where it is thought to have tumor suppressor properties. *FRMD6* may help mediate the process by which Vitamin D inhibits the proliferation of immune cells[58, 59]. Upregulation of *FRMD6* has been suggested as a prognostic marker in colorectal cancer[59]. *KIR2DL4* codes for transmembrane glycoproteins expressed by natural killer (NK) cells and subsets of T cells. *KIR2DL4* inhibits the activity of NK cells and may reduce activation induced cell death in these T cells in Sézary syndrome[60],[61, 62]. *KIR2DL4* is an unusual member of the KIR family that recognizes human leukocyte antigen G and mediates NK-cell activation [63] and has been suggested as a useful diagnostic biomarker of neoplastic NK-cell proliferations[64].

#### Hypothetical mechanisms of down-regulated genes in non-improvement of SOFA score and MELD-XI score and year 1 non-survivors

*SAP25* is a member of the nucleocytoplasmic shuttling proteins that are located in promyelocytic leukemia (PML) nuclear bodies. PML nuclear bodies are implicated in diverse cellular functions, such as gene regulation, apoptosis, senescence, DNA repair, and antiviral response[65],[66, 67]. *NAPSA* is a pronapsin gene, which may have a considerable diagnostic value as a marker for primary lung cancer. NAPSA was detected in a subset of poorly differentiated papillary thyroid carcinomas and anaplastic carcinomas[68]. *TIMP3* is an extracellular matrix-bound protein, which regulates matrix composition and affects tumor growth. *TIMP3* suppresses tumor inactivation in cancer by mechanisms of invasion and angiogenesis[69]. *TIMP-3* downregulation is associated with aggressive non-small cell lung cancer and hepatocarcinoma cells, as compared with less invasive and/or normal lung and liver cells[70]. It mediates vascular endothelial growth factor (VEGF) by blocking the binding of VEGF to VEGF receptor-2, inhibiting downstream signaling, and prevents angiogenesis. These inhibitive properties seem to be independent of its matrix metalloproteinases (MMP)-inhibitory activity, which indicates a new function for this molecule. *RHBDD3* is a member of the rhomboid family of proteases that suppresses the activation of dendritic cells (DCs) and production of interleukin 6 (IL-6) triggered by Toll-like receptors. The rhomboid proteins are involved in signaling via the receptor for epidermal growth factor, mitochondrial homeostasis and parasite invasion[71, 72]. *RHBDD3* negatively controls the activation of DCs and maintains the balance of regulatory T cells and TH17 cells by inhibiting the production of IL-6 by DCs, thus contributing to the prevention of autoimmune diseases[72].

In summary, our central postulate is that OD and death after MCS- or HTx-surgery results from innate and adaptive immune cell dysfunction. Therefore, leukocyte immune-biology information may be used to develop a preoperative test, which more precisely predicts postoperative outcomes in the individual AdHF-patient. To meet this clinical goal, we have developed a novel concept of FRP, which is based on our assessment that the key prognostic information is the preoperative potential to postoperatively restore an equilibrium rather than the absolute magnitude of preoperative OD. In this clinical context, we interpret the potential biological role of the 12 overlap genes as follows: we hypothesize *BATF2* is chronically more activated in GROUP II AdHF-patients in comparison to GROUP I patients. *BATF2* activation is due to its attempts to repair the cell necrosis-mediated damage caused by OD. This hyper-activation leads to exhaustion of adaptive immunity cells, which may explain the protracted time-course-to-death in Group II patients. (Fig 2). To garner support for this hypothesis, we have initiated a study that incorporated multiplex flow cytometry markers, cell free methylated DNA, and mitochondrial DNA into the study protocol. For *RHBDD3*, its downregulation in patients with rheumatoid arthritis, ulcerative colitis and Crohn’s disease[72] may be beneficial in preventing auto-immune aggression. However, its down-regulation in AdHF-patients undergoing MCS-surgery might exacerbate an inappropriate innate inflammatory response and inappropriate adaptive immune-incompetence via a less inhibitory effect on the IL6-pathway[73]. Furthermore, it is interesting to note that upregulation of genes, such as *ANKRD22*, *FRMD6*, and *KIR3DL2*, and down-regulation of genes, such as *TIMP3*, *SAP25*, *NAPSA*, *and TIMP* are associated with a worse prognosis in cancer, are also associated with a worse prognosis in AdHF. This raises the question about common pathways in both clinical syndromes.

#### Health system implication perspectives

Our data suggest that the preoperative dynamic recovery potential, rather than the static severity of OD, is the key prognostic property to restoring equilibrium after surgery. This also presents the possibility of using a preoperative blood sample to identify AdHF-patients who may have a high chance of early postoperative recovery and a potentially good long-term prognosis. If the preoperative blood test result predicts a high FRP (Group I), this data might lead to the recommendation to undergo surgery. If the preoperative blood test suggests a low FRP (Group II), the healthcare team may avoid a potentially harmful recommendation of surgery at that time. In the US, we estimate that out of 30,000–60,000 individuals per year with AdHF and potential candidates for MCS, at least 7,500–15,000 might not benefit from undergoing surgery based on the test results if they are too sick to benefit from MCS surgery. Since HF is a major public health concern due to its tremendous societal and economic burden, with estimated costs in the U.S. of $37.2 billion in 2009 and with expectations to increase to $97.0 billion by 2030, our proposed prediction test would simultaneously allow to tailor the individual patient’s personal benefits and also enhance cost-effectiveness in U.S. healthcare.

The clinical decision-making challenge at the time of AdHF evaluation often culminates in the choice between modern medicine and compassionate end of life care. This ultimate scenario is demanding medically, ethically and economically. It deserves the best evidence-based decision making support that personalized precision medicine research has to offer that lives up to the highest humanistic expectations that society entrusts us with.

#### Limitations

First, our outcome parameter in this proof-of-principle study used a dichotomous endpoint (Improvement versus No Improvement of organ function on day 8 postoperatively). In a planned expansion of the study to include a larger cohort, we will treat the outcome parameter as a quantitative continuous variable. Second, we have not incorporated multisystem level protein markers into our analysis. In a planned extension of the project, we will include multiplex flow cytometry and cytokine parameters. Third, the study had a small sample size. This poses inherent limitations on Group I vs Group II comparisons. The logistic regression/Cox-PH models were constructed with only one predictor variable each due to sample size constraints. We also reported the coefficients/accuracy measures from these models with 95% confidence intervals, which properly reflect our uncertainty about the parameter estimates as a function of sample size. We have reiterated the small sample size of our study, which represents preliminary work to establish feasibility and the need for follow-up/confirmation studies to validate our findings. Fourth, the RT-qPCR validation was limited by a lack of biological material necessary to complete the test. We will expand this validation to include all candidate genes in a follow-up study. Fifth, as in translational biomarker development in general, many results were a consequence of operator/researcher-dependent decisions. Sixth, while we chose to base our analysis on AdHF-patients undergoing MCS-surgery alone to address the problem of MCS-related perioperative MOD[4, 33, 34], we acknowledge that we have not addressed aspects of the PBMC-biology related to MCS-surgery intervention versus general heart surgery. In order to address this question, we have initiated a follow-up project examining AdHF-cohorts undergoing OMM, HTx, coronary artery bypass surgery, percutaneous coronary interventions, valve replacement, valve repair, arrhythmia interventions and healthy volunteers, utilizing the same study protocol. These results will be reported separately.

## Conclusions

In AdHF patients undergoing MCS implantation, the postoperative clinical improvement of OD within one week of surgery is associated with reduced long-term mortality and a PBMC GEP that differs from that of patients who do not improve, is already present preoperatively and may lend itself to outcome prediction. The underlying mechanisms and prognostic implications to improve patient outcomes warrant further study in larger longitudinal cohorts.
